# Autoimmune Neurogenic Dysphagia

**DOI:** 10.1007/s00455-021-10338-9

**Published:** 2021-07-05

**Authors:** Panos Stathopoulos, Marinos C. Dalakas

**Affiliations:** 1 First Department of Neurology, National and Kapodistrian University of Athens Medical School, Athens, Greece; 2 Neuroimmunology Unit, Department of Pathophysiology, Faculty of Medicine, National and Kapodistrian University of Athens, Athens, Greece; 3 Department of Neurology, Thomas Jefferson University, Philadelphia, USA

**Keywords:** Dysphagia, Deglutition, Deglutition disorders, Neurological autoimmunity, Inflammatory myopathies, Myasthenia gravis, Autoimmune neuropathies, Neuromyelitis, Stiff-person syndrome, Immunotherapies

## Abstract

Autoimmune neurogenic dysphagia refers to manifestation of dysphagia due to autoimmune diseases affecting muscle, neuromuscular junction, nerves, roots, brainstem, or cortex. Dysphagia is either part of the evolving clinical symptomatology of an underlying neurological autoimmunity or occurs as a sole manifestation, acutely or insidiously. This opinion article reviews the autoimmune neurological causes of dysphagia, highlights clinical clues and laboratory testing that facilitate early diagnosis, especially when dysphagia is the presenting symptom, and outlines the most effective immunotherapeutic approaches. Dysphagia is common in inflammatory myopathies, most prominently in inclusion body myositis, and is frequent in myasthenia gravis, occurring early in bulbar-onset disease or during the course of progressive, generalized disease. Acute-onset dysphagia is often seen in Guillain–Barre syndrome variants and slowly progressive dysphagia in paraneoplastic neuropathies highlighted by the presence of specific autoantibodies. The most common causes of CNS autoimmune dysphagia are demyelinating and inflammatory lesions in the brainstem, occurring in patients with multiple sclerosis and neuromyelitis optica spectrum disorders. Less common, but often overlooked, is dysphagia in stiff-person syndrome especially in conjunction with cerebellar ataxia and high anti-GAD autoantibodies, and in gastrointestinal dysmotility syndromes associated with autoantibodies against the ganglionic acetyl-choline receptor. In the setting of many neurological autoimmunities, acute-onset or progressive dysphagia is a potentially treatable condition, requiring increased awareness for prompt diagnosis and early immunotherapy initiation.

## Introduction

Dysphagia, manifested as difficulty swallowing solids or liquids, is a symptom of variable severity, from mild to severe, which affects quality of life and may lead to life-threatening complications including malnutrition, weight loss, and aspiration pneumonia. Swallowing is a complex mechanism, requiring the coordination of both skeletal and smooth muscles and the involvement of the central, peripheral, and autonomic nervous system that works in synchrony to coordinate the different phases (oral, pharyngeal, and esophageal). Although dysphagia is commonly caused by a local oropharyngeal or a generalized, systemic medical condition, it can be entirely due to a neurological disease such as stroke, head injury, dementia, and Parkinson’s disease, cerebral palsy, or motor neuron disease (Amyotrophic Lateral Sclerosis). In addition, dysphagia can be the first or sole manifestation of an autoimmune neurological disease, or occurs within the complex of symptoms in the course of many neurological autoimmunities. Since treatment of dysphagia depends on the causative process, identifying an autoimmune trigger or association is critical, because patients with autoimmune dysphagia can respond to immunotherapies. Autoimmune neurogenic dysphagia is, therefore, an important and rather underestimated category that deserves special attention by all specialties.

Autoimmune causes of dysphagia can be gastroenterological, such as IgG4-related disease and eosinophilic esophagitis; dermatological, such as pemphigus vulgaris and bullous pemphigoid; rheumatologic, such as scleroderma, Sjogren’s syndrome, systemic lupus erythematosus, rheumatoid arthritis, Behcet disease, ANCA-associated vasculitis, or granulomatosis with polyangiitis; and neurologic. In this opinion article, we review the autoimmune neurological causes of dysphagia, highlight clinical clues and laboratory tests that facilitate early diagnosis, especially when dysphagia is the presenting symptom, and outline the most effective immunotherapeutic approaches. We focus on common autoimmune neurological diseases, such as inflammatory autoimmune myopathies, myasthenia gravis, autoimmune cranial neuropathies in the spectrum of Guillain–Barre syndrome and multiple sclerosis, and less common like neuromyelitis optica, Stiff-person spectrum disorders and ganglionic acetyl-choline receptor autoantibody-related autoimmunities.

## Autoimmune Neurological Disorders with Dysphagia

Autoimmune neurogenic dysphagia can be seen with diseases affecting muscle, neuromuscular junction, cranial nerves, brainstem, or corticospinal CNS tracts as the first manifestation of an autoimmune process or during the course of a progressive neurological autoimmune disease. The most common disorders in this category, listed in Table 1 with anatomic sequence, include inflammatory myopathies; neuromuscular junction disorders; autoimmune cranial neuropathies; autoimmune ganglionopathies or dysautonomic disorders; and autoimmune central nervous system diseases.

**Table 1:** Autoimmune neurological disorders with dysphagia

A. Inflammatory myopathies
1. Dermatomyositis (DM)
2. Polymyositis (PM)
3. Anti-synthetase syndrome-Overlap Myositis (Anti-SS-OM)
4. Immune-mediated necrotizing myopathy (IMNM or NAM)
5. Inclusion Body Myositis (IBM)
B. Neuromuscular junction disorders
1. Myasthenia gravis (MG)
2. Lambert-Eaton myasthenic syndrome (LEMS)
C. Autoimmune cranial neuropathies
1. Guillain Barre syndrome (GBS) and variants
2. Autoimmune paraneoplastic neuropathies
3. Neuropathies in systemic autoimmune illnesses
D. Autoimmune ganglionopathies and autonomic nervous system disorders
E. Autoimmune centran nervous system disorders
1. Multiple sclerosis (MS)
2. Neuromyelitis optica spectrum disorders (NMO-SD)
3. Glutamic acid decarboxylase (GAD) autoantibody-associated disorders
4. Other autoimmune encephalitides

### Inflammatory Myopathies

The inflammatory myopathies constitute a heterogeneous group of muscle diseases originally classified to include polymyositis (PM), dermatomyositis (DM), and inclusion body myositis (IBM) [1, 2] but now evolved to also include immune-mediated necrotizing myositis (IMNM) or necrotizing autoimmune myositis (NAM), and the anti-synthetase syndrome-overlap Myositis (Anti-SS-OM) [3, 4]. Dysphagia occurs in all subtypes but in our experience, it is especially prominent in IBM, followed by Anti-SS-OM, DM, and NAM.

Despite the heterogeneity among the group of inflammatory myopathies, several studies have assessed prevalence of dysphagia in pooled cohorts of different disease subsets. A meta-analysis, including 116 studies and 10,382 subjects, found that 36% of patients with inflammatory myopathy had dysphagia [5]. Of note, when only studies with low chances of bias were included, the prevalence of dysphagia rose to 82% underscoring the need for high degree of suspicion in early identification. Risk factors for dysphagia were the diagnosis of IBM, an underlying malignancy, or a suspected malignancy as in DM patients with anti-nuclear matrix protein 2 (NXP2) antibodies [5]. A retrospective chart-review study found that 23% among 230 DM/PM patients had dysphagia at disease presentation, but as the disease progressed 58% of the patients developed dysphagia [6]. A second, smaller, retrospective, questionnaire-based study of 50 patients (35 with DM and 8 with PM), found dysphagia at diagnosis in 10% of the patients [7]. Better insight about the prevalence of dysphagia at disease onset or throughout the course of the disease is provided by studies that focus on specific inflammatory myopathy subsets as discussed below. In our overall experience with many inflammatory myopathy patients, dysphagia occurs during the course of the disease and not at presentation, with the exception of IBM where it can be an early symptom generating diagnostic challenges.

#### Dermatomyositis

Dermatomyositis (DM) occurs in children and adults and presents with characteristic skin manifestations accompanying or preceding muscle weakness. Periorbital heliotrope (blue-purple) rash with edema, erythematous rash on face, knees, elbows, malleoli, neck, anterior chest (in V-sign), back and shoulders (in shawl sign), and knuckles with a violaceous eruption (Gottron’s rash) that evolves into a scaling discoloration, are typical skin lesions. Muscle histology shows inflammation predominantly perivascularly, in the interfascicular septae or at the periphery of the fascicle with characteristic perifascicular atrophy and abnormalities of the capillaries [2]. In DM, there is early activation of complement with the C5b-9 membranolytic-attack complex deposited on the endothelial cells leading to capillary necrosis, reduction of endomysial capillaries, ischemia, and muscle fiber destruction resembling microinfarcts [1–3]. In addition, autoantibodies against melanoma differentiation-associated protein 5 (MDA5), anti-transcription intermediary factor 1-gamma (anti-TIF1-gamma), anti-NXP2, and anti-small ubiquitin-like modifier-activating enzyme (anti-SAE) can be present. Among these antibodies, anti-NXP2 that often denotes an underlying malignancy, may correlate with dysphagia [8]. About 15% of DM patients may overall have an underlying malignancy. Dermatomyositis may also co-exist with other systemic autoimmunities, most often systemic sclerosis which by itself is associated with dysphagia due to esophageal fibrosis [9].

Dysphagia in DM ranges from 31% (*N* = 3274) in a rather heterogeneous meta-analysis, to 43% (*N* = 949) in the Euromyositis registry, and even over 60% (*N* = 117) in a cohesive retrospective cohort [5, 6, 10]. In a more targeted retrospective investigation of patients with inflammatory myopathies and dysphagia that also included video-fluoroscopic swallow studies (VFSS), dysphagia was rarely the presenting symptom of DM [11, 12], an observation consistent with our own experience in large series. In one of those studies, dysphagia was associated with increased mortality, with 5 of 18 dysphagic patients dying within the first year of follow-up; this study is not, however, representative because a number of these patients had malignancy and no details on early diagnosis or immunotherapy initiation were provided [11].

Disease suspicion should be raised by the presence of the characteristic skin lesions often associated with increased levels of creatine kinase (CK). The diagnosis is confirmed with muscle biopsy. Treatment with high-dose steroids and steroid-sparing agents (especially methotrexate, azathioprine, or mycophenolate) is helpful early in the disease. In poorly responding patients, high-dose intravenous immunoglobulin (IVIg) is the treatment of choice [13]. In the only controlled study, IVIg had a dramatic effect on patients’ proximal muscle strength and neuromuscular activities, based on a 20-item neuromuscular symptom score that included dysphagia [13]. Because DM is a complement-mediated microangiopathy, the clinical efficacy of IVIg is immunopathologically supported by inhibition of complement activation and interception of membranolytic-attack complex on endothelial cells, restoring the integrity and population of endomysial capillaries [14]. If IVIg is not sufficiently effective, rituximab is initiated. Rehabilitation measures and compensatory interventions (special diet, feeding techniques, such as small bites and alternating solids and liquids, and exercises such as tongue base retraction and effortful swallow) may provide additional benefits [11]. In severe early cases, percutaneous endoscopic gastrostomy (PEG) may be initially required to prevent aspiration pneumonia and malnutrition until the aforementioned immunotherapies take effect, typically within 2–3 months. A common mistake we have encountered is the prolonged use of PEG that leads to severe atrophy of the pharyngeal and laryngeal muscles and more difficulty returning to normalcy.

#### Polymyositis

Polymyositis (PM) is a very rare entity. In our experience, most patients referred for PM have another muscle disease, most often IBM, NAM, or an inflammatory dystrophy. Based on the aforementioned older studies, dysphagia occurs in 35%-50% of PM patients [5, 6, 10], but these data should be interpreted with caution because it is highly unlikely that these estimates refer to PM; they do, however, reflect that dysphagia is a frequent symptom in inflammatory myopathies.

#### Anti-Synthetase Syndrome-Overlap Myositis

Anti-synthetase syndrome-overlap Myositis (Anti-SS-OM) is now a distinct entity that often presents with systemic sclerosis-like lesions, mild-to-moderate proximal muscle weakness, arthritis in the form of subluxation of the interphalangeal joints, “mechanic’s hands,” Raynaud phenomenon, and interstitial lung disease [3]. The syndrome is highlighted by the presence of various anti-aminoacyl transfer RNA synthetase autoantibodies, primarily anti-Jo-1; hence, its name *“anti-Jo-1 syndrome.”* These patients’ muscle biopsy has distinct findings with necrotizing features in the perimysium and perifascicular muscle fibers [3, 4]. Dysphagia is common (reported in 26% of anti-SS-OM in the Euromyositis registry [10]), but it is clinically mild to moderate and not as severe as in the other inflammatory myopathy subtypes. In a retrospective study of patients with inflammatory myopathies and dysphagia that included VFSS in approximately half the patients, dysphagia was the leading presenting symptom in one of 9 anti-SS-OM patients [11]. Immunotherapy for dysphagia in anti-SS-OM is the same as in DM. In our experience, dysphagia anti-SS-OM patients respond to therapies much better and faster than in DM; this is also corroborated by the 5-year survival data when immunotherapy was applied to nine anti-SS-OM patients with dysphagia as their leading symptom [11].

#### Immune-Mediated Necrotizing Myopathy

Immune-mediated necrotizing myopathy (IMNM) or necrotizing autoimmune myositis (NAM) has now evolved into the most common inflammatory myopathy in all age groups [3]. It starts either acutely, reaching its peak over days or weeks, or subacutely, progressing steadily and causing severe muscle weakness, including dysphagia, and very high (in the thousands) CK levels [3]. NAM may also occur after viral infections and in association with cancer or immune checkpoint inhibitors. Although often attributed to statins or over-diagnosed as a “statin-myopathy” in patients on chronic statin treatment, there is no convincing evidence that statins play a triggering role in patients who develop subacute NAM while taking statins for years [1, 14–16]. Since NAM is now the commonest inflammatory myopathy and more than 25% of Americans above 40 years take statins, the association between statins and NAM is likely a chance phenomenon [15, 16]. Most NAM patients have antibodies against signal recognition particle (SRP) or 3-hydroxy-3-methylglutaryl-coenzyme A reductase (HMGCR), a ubiquitous and non-muscle-specific antigen within the endoplasmic reticulum, more often seen in NAM patients associated with cancer.

Dysphagia was observed in more than 30% of NAM patients in the Euromyositis registry (total *N* = 3067, NAM *N* = 107) [11]. In an in-depth investigation (including VFSS) of five NAM patients with early or prominent dysphagia triggered by immune checkpoint inhibitors (ICPI), only one of four examined patients had cricopharyngeal prominence on VFSS, but all had impaired pharyngeal contraction and three impaired tongue base retraction and epiglottic inversion/laryngeal elevation with aspiration; concurrent ophthalmoparesis was also noted, as commonly observed in ICPI-triggered NAM [12]. In our experience, most NAM patients, even those with severe disease, respond to immunotherapy with intravenously administered corticosteroids in the acute phase, followed by IVIg and, if needed, rituximab.

#### Inclusion Body Myositis

Inclusion Body Myositis (IBM) is the most common and disabling inflammatory myopathy above the age of 50 [1–3] and the most common cause of disabling dysphagia related to inflammatory myopathies. It starts insidiously, over years, at times asymmetrically and progresses steadily simulating a late-life muscular dystrophy or slowly progressive motor neuron disease leading to significant disability [1–3]. Although IBM is commonly suspected when a patient with presumed PM does not respond to therapy [1–3], early involvement of distal muscles, especially foot extensors and finger flexors, atrophy of the forearms and quadriceps muscles, dysphagia, frequent falls due to quadriceps muscle weakness causing buckling of the knees and mild facial muscle weakness are clues to early clinical diagnosis [1–3]. The diagnosis based on the distinct clinical phenotype is confirmed with the muscle biopsy that shows inflammatory infiltrates consisting predominantly of CD8^+^ cytotoxic T-cells invading muscle fibers expressing MHC-I antigen, and autophagic vacuoles with congophilic amyloid deposits [1–3]. Antibodies against cytosolic 5ʹ-nucleotidase 1A (cN1A) are detected in 50% of patients but lack specificity.

Dysphagia is a disabling clinical manifestation of IBM and more frequent than in any other inflammatory myopathy [17–24]. Cumulative dysphagia prevalence was up to 50% in the Euromyositis registry and 56% in a meta-analysis [5, 10], but these studies were quite heterogeneous and not clinically detailed. In several single-center studies with reliable data, dysphagia was the sole presenting symptom or one of the main presenting symptoms in 10% of IBM patients [12, 22, 23], with the frequency rising up to 40% by the time of diagnosis [19]. Targeted in-person questioning of IBM patients revealed dysphagia in 65% (*N* = 57) or up to 80% (*n* = 19); VFSS revealed signs of impaired propulsion in 77% (repetitive swallowing 25%, residues 36%, and cricopharyngeal dysfunction 16%) and aspiration-related signs (mostly inadequate epiglottal downward tilting) in 53% (*N* = 43) [19, 20]. In a study of myopathies with early or prominent dysphagia, 15 patients with IBM were studied with VFSS [12]; an impaired pharyngeal contraction was noted in 9, impaired tongue base retraction in 10, and epiglottic inversion/laryngeal elevation in 8 with their pharyngeal phase more severely affected than the oral phase; aspiration during the study was observed in 3 patients [12]. In a series of 35 IBM patients studied with VFSS, pharyngeal pooling was observed in 85%, impaired tongue base retraction in 76%, and impaired laryngeal elevation in 50%; in 26%, aspiration was noted during the study [11]. A questionnaire in 64 IBM patients followed over a median period of 12 years, found that dysphagia was present in 80% of the 15 survivors [17]. The presence of cricopharyngeal sphincter dysfunction/bar (possibly amenable to intervention) ranged from 42% on barium swallow [19] to 37–47% by VFSS [11, 17, 18]. Not surprisingly, studies focused on dysphagia showed that among all inflammatory myopathies, most patients had IBM [11, 12]. Of importance, isolated dysphagia can be the presenting symptom of IBM [23] or one of the presenting symptoms in 42% of patients [11]. In our experience, progressive dysphagia in IBM is a sign of more aggressive disease, as recently confirmed by others [12].

**Fig. 1:**
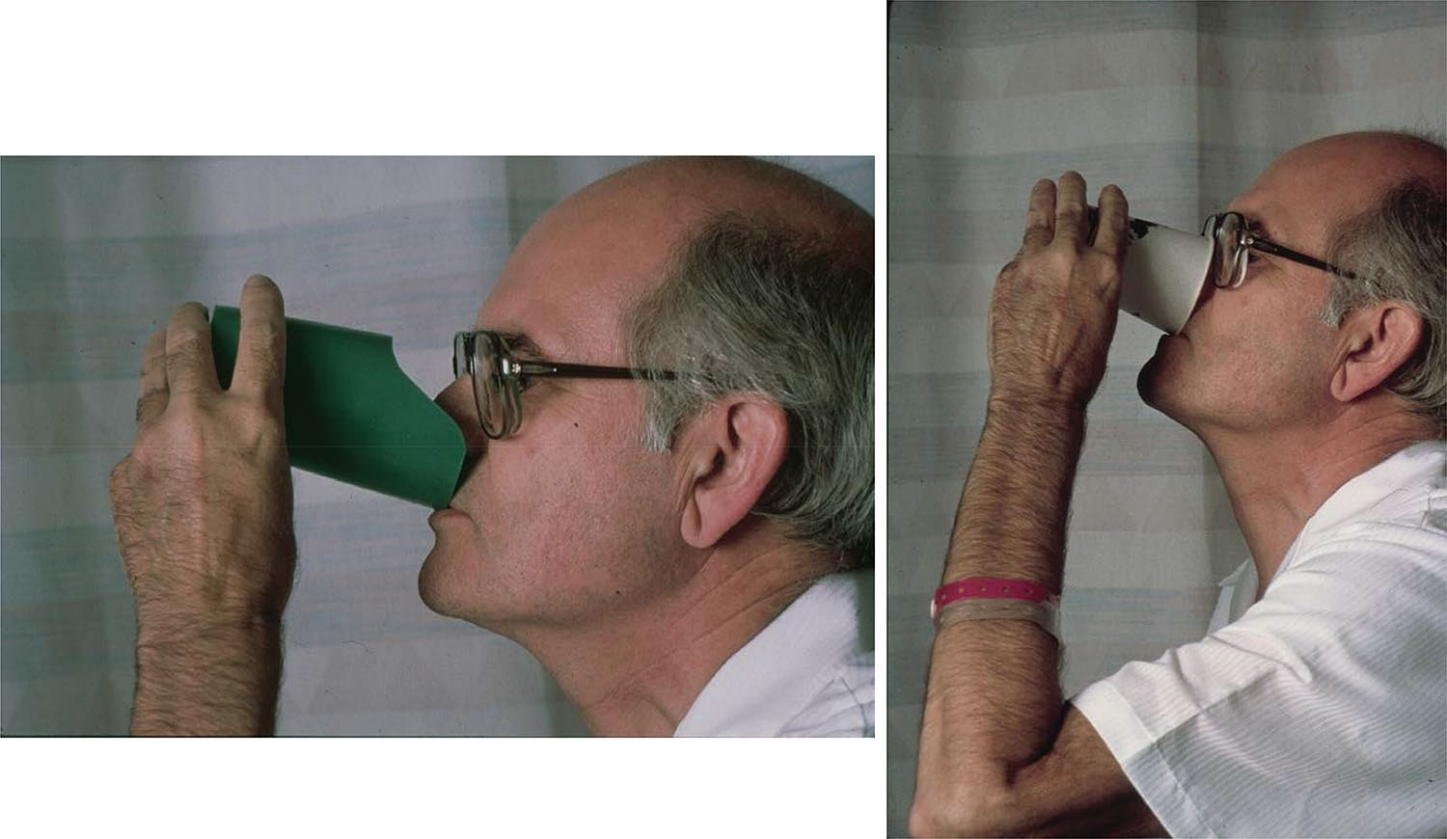
A patient with Inclusion Body Myositis (IBM) participating in an NIH-controlled clinical trial with high-dose intravenous immunoglobulin (IVIG). Left: Before therapy, a patient with swallowing difficulty due to IBM is using a partially cut cup designed by the Swallowing Section at the NIH to raise the cup when drinking without the need to extend the head backwards due to a choking feeling. Right: After IVIg therapy, swallowing improved and patient was able to drink from a normal cup (Dalakas et al. [21])

The only proven treatment that may partially help the dysphagia in some patients with IBM is IVIg based on a controlled study we have conducted [21]. In this study, IVIg did not show significant benefit in muscle strength but did show significant improvement in swallowing, as assessed with VFSS combined with a sensitive quantitative ultrasound method that objectively measures the mean time (in seconds) needed to complete three dry and three wet swallows (Fig. 1 and Table 2) [21]. All immunosuppressive agents used in IBM have failed probably because the disease starts long before patients seek medical advice. Glucocorticoids, methotrexate, cyclosporine, azathioprine, or mycophenolate is ineffective, and although some patients initially experience mild improvements, there is no long-term benefit. Treatment with alemtuzumab, a B and T cell-depleting anti-CD52 monoclonal antibody, has shown some promising results in an uncontrolled trial [25], while treatment with canakinumab, an anti-IL-1β monoclonal antibody, yielded mixed results in a small trial of 5 patients [26]. Anakinra, an IL-1 receptor blocker, had also shown mild short-term improvements in a subset of patients in the first series [27] and in a later study of 15 patients [28]. Because of the common presence of a cricopharyngeal bar, invasive procedures including balloon dilatation and myotomy can lead to improvement of symptoms in patients with serious dysphagia and weight loss prior to PEG insertion [20, 29–33], although this effect was not long lasting after 5 years [17]. In our experience, Botox injections have not been helpful. Regarding rehabilitation techniques, the Mendelsohn maneuver (conscious prolongation of peak-swallow laryngeal elevation) has shown some efficacious results [30]. Overall, although life expectancy has been reported unaffected, most IBM patients with end-stage disease require assistive devices such as cane, walker, or wheelchair [34]; in those patients, dysphagia is the most life-threatening symptom if not responding to IVIg.

**Table 2:** Mean duration (seconds) of ultrasound-recorded swallows at baseline, 3 months, and end of crossover in IBM patients treated with placebo or IVIg

	Type of swallow	Baseline	Three months	Crossover
			Placebo	IVIg
Patients receiving randomly assigned *placebo first* (*n* = 10)	D1	2.33	1.83	1.91
	D2	2.15	1.75	1.88
	D3	1.84^*^	2.84^*^	1.83^*^
	W1	1.59^*^	2.25^*^	1.86^*^
	W2	2.49	1.96	2.00
	W3	1.59^*^	2.07^*^	2.44^*^

			IVIg	Placebo
Patients receiving randomly assigned *IVIg first* (*n* = 9)	D1	3.00^*^	1.62^*^	1.47
	D2	3.37^*^	2.02^*^	2.31
	D3	2.35	2.74^*^	1.85^*^
	W1	1.82	1.86	1.47
	W2	1.73^*^	1.24^*^	1.54
	W3	1.98^*^	1.49^*^	1.48

Reproduced from Dalakas et al. [21]

*D1-3* three dry swallows, *W1-3* three wet swallows, *IBM* inclusion body myositis, *IVIg* intravenous immunoglobulin

^*^p = 0.05

### Neuromuscular Junction Disorders

Myasthenia gravis (MG) is a prototypic autoimmune disease manifesting with skeletal, bulbar, and respiratory muscle weakness, fatigue especially with repetitive movement or muscle actions, and impaired chewing or swallowing [35]. The MG symptoms and pathology are mediated by autoantibodies against nicotinic acetyl-choline receptors (AChR), detected in 85% of patients. The AChR antibodies fix complement at the end-plate region, leading to destruction of the AChRs and simplification of the endplates. In 15% of patients, anti-AChR antibodies are not detected; of those, ~ 50%, comprising 5–8% of all AChR-negative MG cases, are positive for antibodies against muscle-specific kinase (MuSK), a transmembrane polypeptide expressed at the neuromuscular junction that plays a fundamental role in AChR clustering [36]. Anti-MuSK antibodies are of the IgG4 subclass and are typically non-complement binding. MG may have an early or late onset and can remain ocular, when symptoms are limited to extraocular muscles (ptosis, diplopia), or become generalized, when weakness affects most muscle groups. MuSK-MG commonly presents with bulbar symptoms such as dysphagia, dysphonia, dysarthria, and difficulty chewing [37], with dysphagia being more common than in the AChR-MG [38].

Dysphagia in MG (as in all cases of neurogenic dysphagia) manifests with difficulty swallowing both solids and liquids and is primarily caused by pharyngeal and laryngeal muscle weakness and fatigue, and secondarily esophageal hypomotility [39, 40]; the latter is confirmed by esophageal manometry that reveals decreased amplitude of upper esophageal sphincter contractions and esophageal peristaltic waves [41]. Weakness and fatigue can also affect mastication muscles, causing difficulty in chewing. In MG, dysphagia is usually accompanied by other bulbar symptoms such as dysphonia and dysarthria, ocular manifestations like ptosis, and diplopia, and axial or neck muscle weakness [37, 42]. In a large prospective study, clinically assessed dysphagia was present at disease onset in 25% of 84 MG patients [43]. In a study that examined 175 MG patients primarily presenting with head and neck symptoms, clinically assessed dysphagia was the primary symptom in 15% and the secondary in 12% [44]. Dysphagia can also be part of the clinical syndrome in another autoimmune neuromuscular junction disorder, the Lambert-Eaton Myasthenic Syndrome (LEMS), which can be paraneoplastic in about 50–75% of the patients, and is associated with antibodies against the P/Q type voltage-gated calcium channels (VGCC) at the presynaptic nerve terminals [45].

The diagnosis of MG is solidified by testing the presence of serum AChR or MuSK autoantibodies. In the 7% of seronegative MG, the diagnosis, if clinically suspected based on fatiguing weakness, is confirmed with single-fiber EMG or repetitive nerve stimulation studies. Dysphagia related to seronegative MG should be distinguished, when advanced, from ALS, where there is also associated tongue atrophy. Electromyographic testing clarifies the diagnosis but if in doubt, a trial with oral pyridostigmine 60 mg QID can be helpful. Identifying MG-related dysphagia is clinically rewarding for the clinicians and the patients because today MG is a treatable disease [46–48]. After excluding thymoma—seen in 10% of all AChR-MG patients-, the AChR-positive MG patients respond to pyridostigmine (an acetyl-cholinesterase inhibitor) followed by steroids and a steroid-sparing immunosuppressant such as azathioprine or most preferably today mycophenolate. Difficult cases, or when in a crisis and the dysphagia is associated with breathing difficulties, respond to IVIg and plasmapheresis [49]. Rituximab, an anti-CD20 + monoclonal antibody leading to B-cell depletion from the circulation [46, 47], is also effective in the chronic management of refractory cases. In MuSK-MG, where the antibodies are of the IgG4 subclass, rituximab is a preferred drug even early in the disease course, leading to impressive long-term remission. It is imperative, therefore, to identify early, at symptom onset, if dysphagia is due to a specific MG subtype because it is a treatable condition with the suitable agents in the majority of patients. Therapy in MG today is a success story because in addition to the aforementioned agents, new drugs, such as eculizumab, a monoclonal antibody against complement C5, are now approved for severe or refractory MG. Another new agent directed against the neonatal Fc receptor (FcRn), which enhances the catabolism of circulating IgG antibodies, was effective in phase III trials and is in the process of gaining approval [46].

### Autoimmune Cranial Neuropathies

Cranial neuropathies of autoimmune origin can also cause dysphagia, either in isolation or together with other symptoms associated with the same or neighboring nerves. Horizontal binocular diplopia (cranial nerve VI), facial paralysis, and dysarthria (cranial nerve VII), impaired hearing, balance, vertigo and tinnitus (cranial nerve VIII), impaired taste and sensation in the posterior tongue and palate (cranial nerve IX), nasal voice/hoarseness (cranial nerve X), paresis of the trapezius and sternocleidomastoid (cranial nerve XI), and dysarthria (cranial nerve XII) are among the accompanying symptoms. In the case of cranial neuropathies, the pathomechanism of dysphagia involves abnormal signal conduction to the swallowing muscles primarily affecting the pharyngeal and laryngeal muscle groups (innervated by cranial nerves IX-X), thus, causing dysphagia of both liquids and solids. Additionally, cranial nerve XII that innervates the tongue muscles, cranial nerve VII that innervates the lip muscles, and cranial nerve V that innervates the muscles of mastication, contribute to the oral phase of swallowing. Importantly, cranial nerve X carries autonomic fibers to the gastrointestinal system and influences esophageal motility. The most common autoimmune/inflammatory neurological diseases that affect cranial nerves resulting in dysphagia are as follows:

#### Guillain–Barre Syndrome (GBS) and Its Variants

Guillain–Barre syndrome (GBS) constitutes a monophasic, often postinfectious, autoimmune polyradiculoneuropathy that manifests acutely or subacutely (in less than 4 weeks). Typically GBS presents with ascending weakness, but in the Miller Fisher syndrome (MFS) and the pharyngeal-cervical-brachial (PCB) variant, cranial and cervical nerves are predominantly affected. The MFS variant typically presents with the triad of ataxia, ophthalmoplegia, and areflexia, while the PCB variant with cervicobrachial and oropharyngeal weakness including dysphagia [50–52]. The GBS, PCB, and MFS, as well as Bickerstaff’s encephalitis, are viewed as a continuum of the same syndrome [51–53] with dysphagia even being the presenting symptom in isolated cases [54]. Notably, MFS is associated with anti-GQ1b and PCB with anti-GT1a antiganglioside autoantibodies with significant cross-reactivity [51, 52, 55]. In eight patients with anti-GT1a antibodies, clinical dysphagia was noted in 88% of cases [56]. In GBS, prevalence of clinical dysphagia ranges from 41% (*N* = 54, [56]) to 53.5% (*N* = 71, [57]), being higher (75%, *N* = 16) in patients admitted to ICU [58]. When dysphagia was assessed by VFSS in 14 GBS patients referred for swallowing evaluation, abnormalities were present in all the patients, with the pharyngeal swallowing phase being more severely affected than the oral phase; among these patients, 6 had equal involvement of oral and pharyngeal phases, 7 had more severe involvement of the pharyngeal phase, and one more severe involvement of the oral phase) [59]. Electrophysiological swallow studies also revealed silent dysphagia in 28% of non-ICU admitted GBS patients, while 38% of patients in that cohort had clinically overt dysphagia [60]. These studies collectively underline that clinicians need to remain vigilant and maintain a low threshold for nasogastric tube placement in GBS patients.

The diagnosis of GBS, MFS, and PCB is facilitated by history of preceding viral or bacterial infection, like Zika virus, SARS-CoV-2 or Campylobacter jejuni [61, 62], the presence of areflexia on examination, elevated protein in an acellular CSF, possible nerve root enhancement on MRI, and signs of neuropathy (mostly demyelinating, but sometimes axonal) on EMG. Prompt diagnosis is of importance as IVIg and PLEX are equally effective and should be initiated as soon as possible [63, 64]. In chronic inflammatory demyelinating polyradiculoneuropathy, which is the chronic counterpart of GBS and has subacute onset or a relapsing course [65, 66], dysphagia is uncommon but, if occurs, it responds to immunotherapy with IVIg, steroids, or rituximab in pace with the other symptoms [67–70].

#### Autoimmune Paraneoplastic Neuropathies

Autoimmune paraneoplastic neuropathies are caused by immunological consequences, triggered by malignant tumors targeting the nerves including those responsible for swallowing. They should be distinguished from direct infiltration of cranial nerves by a tumor, like lymphoma [71, 72], and by chemotherapy or radiation-associated neuropathies. Autoimmune neuropathies in a setting of cancers can be also triggered by immune checkpoint inhibitors that aberrantly stimulate the immune system inducing various immunological syndromes affecting nerve, muscle, neuromuscular junction, and brain [72, 73]. Typically, but not always, the autoimmune process is demonstrated by the presence of autoantibodies [74, 75]. Paraneoplastic autoimmune neuropathies causing dysphagia, although not common, are important to recognize not only because they can precede the tumor diagnosis, but also because they can respond to immunotherapy and tumor removal [74, 76, 77]. Two antibodies in patients with small cell lung cancer, the anti-Hu (antineuronal nuclear antibody-1), [78, 79] and anti-Ri (antineuronal nuclear antibody-2) may be markers of paraneoplastic neuropathy with more prominent dysphagia [80]. In a study of 34 patients with Ri autoantibodies, clinical dysphagia was observed in seven, in conjunction with polyradiculoneuropathy, brainstem, and cerebellar involvement or encephalopathy [80]. Treatment of paraneoplastic autoimmune neuropathies consists of tumor removal and initiation of immunotherapy. The anti-Hu syndrome, in particular, can be aggressive not well-responding immunotherapies, although some cases have responded to cyclophosphamide [76].

#### Neuropathies in Systemic Autoimmune Diseases

A systemic disorder where cranial neuropathies are prominent is sarcoidosis, where in 5–10% of cases, they are the presenting symptom [81]. The most common cranial neuropathy in sarcoidosis is the one affecting the facial nerve (VII), but other nerves including the IX and X can be affected, resulting in dysphagia. Of note, involvement of other organs, such as the esophagus [82, 83] and the swallowing muscle apparatus [84], can also cause dysphagia. Diagnosis is aided by increased circulating ACE levels, chest CT showing hilar lymphadenopathy, brain MRI showing leptomeningeal enhancement, PET-CT showing sites of active inflammation, lumbar puncture revealing high protein and oligoclonal bands, and ultimately biopsy of accessible tissue showing non-caseating granulomas. Sarcoidosis typically responds to corticosteroids and steroid-sparing agents such as mycophenolate, and in more severe cases to anti-TNF agents, such as infliximab, or anti-CD20 agents like rituximab [82–84]. Neuropathy can also occur in the course of Sjogren’s syndrome (SS) [85, 86], systemic lupus erythematosus [87], and rheumatoid arthritis [88]; among these disorders, dysphagia is more prominent in SS [89], even though sometimes it is erroneously attributed to lack of saliva and esophageal hypomotility rather than involvement of the cranial nerves [90].

### Autoimmune Ganglionopathies and Autonomic Nervous System Disorders

In rare instances, an autoimmune response can target the dorsal root ganglia as well as elements of the autonomic nervous system that control the gastrointestinal tract [91]. Such syndromes manifest with widespread dysautonomia including gastrointestinal dysmotility and dysphagia, and can be associated with antibodies against the ganglionic AChR (gAChR). In cases of paraneoplastic association, antibodies to Hu antigen or to neuronal voltage-gated calcium channel (VGCC), mainly the N-type and to a lesser degree the P/Q type (also associated with LEMS), can be seen. In the presence of gAChR, and to a lesser degree VGCC autoantibodies, dysphagia can be directly caused by esophageal motility disorders, primarily achalasia and rarely diffuse esophageal spasm [91, 92]. Once diagnosis of an autoimmune cause is established, search for an underlying neoplasia is warranted and, accordingly, tumor removal and/or initiation of immunotherapy.

### Autoimmune CNS Disorders

Brain regions important for swallowing localize to the anterior insula and the frontoparietal operculum, reaching via corticobulbar fibers, the brainstem nuclei of cranial nerves V, VII, XII, IX, X involved in the oral, pharyngeal, and esophageal phases of swallowing ([]. Brainstem lesions account for the majority of cases of CNS dysphagia, followed by insular and opercular lesions. Autoimmune CNS disorders causing dysphagia include multiple sclerosis (MS), Neuromyelitis Optica Spectrum Disorders (NMO-SD), hyperexcitability disorders within the spectrum of stiff-person syndrome, and autoimmune encephalitis.

#### Multiple Sclerosis

In Multiple sclerosis (MS), dysphagia is most often seen in primary or secondary progressive forms and correlates with the expanded disability status scale (EDSS), highlighting its association with advanced stages of the disease [93–95]. Dysphagia can be, however, seen in some acute MS cases when active demyelinating lesions affect the brainstem, and in rare cases, when active lesions affect the operculum [96]. In studies with more than 200 MS patients, where dysphagia was either evaluated clinically or by questionnaires, the prevalence of dysphagia ranged from 21 to 43% [96–103]. In studies that employed objective assessments, like VFSS and fiber endoscopic swallow studies (FESS), the dysphagia prevalence ranged from 54 to 90% [104–107] highlighting that MS clinicians should maintain a low threshold for suspecting swallowing disturbances, especially in patients with progressive MS and high EDSS scores. A VFSS study in 18 MS patients (10 with symptomatic dysphagia of varying degree and 8 asymptomatic) demonstrated aspiration in all symptomatic patients with undercoating of the epiglottis and/or laryngeal penetration; the VFSS was also abnormal in 6 of the 8 asymptomatic patients [106]. Diagnosis of MS is based on clinical, radiological, and CSF characteristics of relapses or progression and exclusion of alternative diagnoses [108, 109]. Early immunotherapy with high-efficacy treatments decreases the risk for secondary disability progression [109, 110]. In addition to immunotherapy, rehabilitation measures such as dietary modifications, compensatory techniques, and training by a speech therapist are suggested. The value of more invasive techniques, such as Botox injection to an overactivated cricopharyngeal muscle, warrant further study [96–98, 111].

#### Neuromyelitis Optica Spectrum Disorders

The NMO-SD are defined, apart from optic neuritis and myelitis, by antibodies against aquaporin 4 (AQP4) [112] and myelin oligodendrocyte glycoprotein (MOG) [113]. In AQP4-NMO-SD, clinical dysphagia can be most often present in combination with other symptoms such as nausea, vomiting, intractable hiccups, dysarthria, or hypoglossal palsy [114, 115] but can rarely occur in isolation [116, 117]. Dysphagia is typically caused by a lesion in the brainstem and medulla oblongata and may involve the nucleus ambiguus, located close to the area postrema, which is typically affected in AQP4 NMO-SD and is responsible for the intractable hiccups, nausea, and vomiting [117, 118]. The area postrema is a circumventricular area that allows access to serum AQP4 autoantibodies, even with intact blood brain barrier [119, 120].

In a cohort of 170 NMO-SD patients (most AQP4-autoantibody positive), medulla oblongata lesions were present in 26% of patients, with 30% of them having clinical dysphagia or choking cough. In contrast, dysphagia was rare in patients without medulla oblongata lesions (noted in 1.4%) [121, 122]. Clinical dysphagia can also occur in seronegative or MOG autoantibody-positive NMO-SD, again in connection with brainstem involvement.

Studies with FESS in 13 NMO-SD patients (6 with AQP4, 5 with MOG autoantibodies, and 2 seronegative) revealed abnormalities in 8 [124]. Interestingly, 6 of them, classified as mildly dysphagic by FESS (compared to controls), were clinically asymptomatic without MRI signs of brainstem involvement; the patients with moderately or severely dysphagia by FESS had, however, brainstem involvement. The study points out that subclinical dysphagia can be frequently present in NMO-SD, possibly indicating brainstem damage not shown by MRI, as also noted above for some MS patients [124]. Such subclinical dysphagia, identified by ultrasound, has been also seen in patients with post-polio syndrome due to late motor neuron dysfunction in the brainstem nuclei, 20–30 years after acute paralytic poliomyelitis; of interest, autoimmunity has been implicated in these patients leading to an ongoing trial with intravenous immunoglobulin [123].

Diagnosis in NMO-SD is greatly aided by testing for AQP4 and MOG autoantibodies. Optimal treatment of all symptoms, including dysphagia, involves immediate and aggressive immunotherapy with corticosteroids, plasma exchange, or IVIg, followed by maintenance therapy with mycophenolate [124], rituximab [125], inebilizumab [126], or eculizumab [127]. Similar therapies, steroids for acute relapses [128] and B-cell depletion therapies [129, 130], are also applied in MOG-positive cases.

#### Stiff-Person Syndrome (SPS) and GAD-Spectrum Disorders (GAD-SD)

The presence of high-titer antibodies against glutamic acid decarboxylase (GAD) in the patients’ serum, also reflecting intrathecal synthesis, defines the SPS-spectrum disorders (SPS-SD or GAD-SD) that include stiff-person syndrome, cerebellar ataxia, autoimmune epilepsy, and encephalopathy [131–133]. SPS is clinically characterized by stiffness and muscle spasms in the trunk and proximal limbs that often include the facial muscles; 15% of patients also have ataxia, dysarthria, and dysphagia. Part of the SPS-SD is also Progressive Encephalopathy with Rigidity and Myoclonus (PERM), a distinct syndrome characterized by muscle stiffness, spasms, myoclonus, and brainstem dysfunction with oculomotor abnormalities, dysphagia, gait ataxia, prominent autonomic involvement, and the presence of anti-Glycine receptor antibodies. Although GAD antibodies have not been demonstrated to be pathogenic, the anti-Glycine receptor antibodies seem to be [134, 135]. Dysphagia can be present in both PERM and SPS, especially when bulbar, brainstem, or cerebellar symptoms are prominent as seen in at least 15% of patients [132–134]. In our experience with a large number of SPS-SD, dysphagia can be a debilitating symptom, especially in patients with co-existing cerebellar ataxia and is often accompanied by dysphonia.

The treatment of dysphagia in SPS-SD and PERM includes symptomatic therapies with GABA-enhancing drugs, such as benzodiazepines, baclofen or gabapentin, and immunotherapy with IVIg or rituximab [131]. In a controlled trial of SPS patients, IVIg was effective and is now the treatment of choice [136]. A controlled trial with rituximab did not, however, show statistically significant differences owing to a strong placebo effect, but a subset of SPS patients exhibited strong and sustained improvements [137].

#### Autoimmune Encephalitides

In autoimmune encephalitides, dysphagia can be present, although this association is often confounded by impaired level of consciousness. Clinical dysphagia has been reported in several acute encephalitides including (a) those with antibodies to synaptic antigens such as NMDAR and Gamma Aminobutyric Acid receptor B (GABAR-B) antibodies; (b) acute disseminated encephalomyelitis (ADEM) as part of a bilateral opercular syndrome, also described as Foix–Chavany–Marie syndrome, where paralysis of facial, tongue, pharyngeal, and masticatory muscles can be prominent [138, 139] (c) Chronic Lymphocytic Inflammation with Pontine Perivascular Enhancement Responsive to Steroids (CLIPPERS) [140, 141]; (d) Dipeptidyl Peptidase-like Protein-6 (DPPX)-autoantibody-associated disease with prominent gastrointestinal dysmotility [142]; (e) Hashimoto encephalopathy [143]; (f) Bickerstaff brainstem encephalitis [144, 145], and (g) the recently identified disease characterized by autoantibodies against the brain neuronal adhesion molecule IgLON5, which presents with a bulbar syndrome that includes (as often as 50–86% of cases) dysphagia, along with sleep disturbances (parasomnias, difficulty breathing), cognitive decline, and progressive supranuclear palsy-like symptoms [146, 147]. Dysphagia can be also seen within the clinical spectrum of several, predominantly paraneoplastic, autoimmune CNS syndromes, characterized by autoantibodies against intracellular antigens such as Hu, Ri, neurochondrin, Glial Fibrillary Acidic Protein (GFAP), Ma antigen-2 (Ma2), or Rho GTPase-activating protein 26 [80, 148–151]. In all those entities dysphagia treatment includes immunotherapy, which can be variably effective.

## Summary and Conclusion

Dysphagia is part of the clinical spectrum of various autoimmune neurological disorders affecting the whole neuraxis (muscle, neuromuscular junction, nerve, roots, brainstem and cortical regions), occurring either alone or in combination with other symptoms. Awareness of the causative factors and early clinical manifestations of autoimmune neurogenic dysphagia are important because prompt initiation of immunotherapy is an effective means of alleviating dysphagic symptoms. Dysphagia can be manifested insidiously in the presence of an existing autoimmune neurological disease, but it can also present acutely or subacutely as the early manifestation of an evolving neuro-autoimmunity. In inflammatory myopathies, dysphagia most commonly occurs in IBM, but it is not uncommon in DM, anti-SS-OM, and NAM/IMNM; an elevated CK raises suspicion, and a muscle biopsy confirms the diagnosis. While immunotherapy is generally effective, IBM remains still difficult to treat. In MG, dysphagia is often associated with fatigability and other cranial nerve involvement; when diagnosis is confirmed by detection of specific autoantibodies, dysphagia has an impressive response to immunotherapy. In autoimmune neuropathies, acute-onset dysphagia as seen in PCB, MFS and GBS variants, should be recognized and treated early to optimize outcome; more insidious dysphagia is seen in paraneoplastic neuropathies, highlighted by anti-Hu/-Ri autoantibodies and in ganglionopathies associated with autonomic dysfunction, gastrointestinal dysmotility, and antibodies to gAChR or VGCC. In paraneoplastic syndromes, although symptoms can be more difficult to treat, response to immunotherapy is variable but more promising in the presence of pathogenic autoantibodies against surface proteins. Among the CNS causes of autoimmune neurogenic dysphagia are demyelinating and inflammatory lesions of the brainstem, usually in the setting of advanced MS and NMO-SDs associated with autoantibodies against AQP4 and MOG, where early diagnosis is of paramount importance as response to immunotherapy can be rewarding. Autoimmune hyperexcitability disorders, highlighted by the SPS-SDs that typically present with hyperexcitability, spasms, and high-titer anti-GAD autoantibodies, are overlooked causes of potentially treatable autoimmune dysphagia. Considering the multitude of neurological autoimmune disorders and their disease mimics causing dysphagia, increased awareness is critical to establish the correct diagnosis and initiate immunotherapy as most of the underlying neurological disorders are potentially treatable.

## Abbreviations

CNS: Central nervous system
GAD: Glutamic acid decarboxylase
IgG4: Immunoglobulin G subclass 4
ANCA: Antineutrophil cytoplasmic antibody
PM: Polymyositis
DM: Dermatomyositis
IBM: Inclusion body myositis
IMNM: Immune-mediated necrotizing myositis
NAM: Necrotizing autoimmune myositis
Anti-SS-OM: Anti-synthetase syndrome-overlap myositis
NXP2: Anti-nuclear matrix protein 2
C5b-9: Complement component 5b-9
MDA5: Melanoma differentiation-associated protein 5
Anti-TIF1-gamma: Anti-transcription intermediary factor 1-gamma
anti-SAE: Anti-small ubiquitin-like modifier-activating enzyme
VFSS: Video-fluoroscopic swallow studies
CK: Creatine kinase
IVIg: Intravenous immunoglobulin
PEG: Percutaneous endoscopic gastrostomy
SRP: Signal recognition particle
HMGCR: 3-Hydroxy-3-methylglutaryl-coenzyme A reductase
ICPI: Immune checkpoint inhibitors
CD: Cluster of differentiation
MHC: Major histocompatibility complex
MG: Myasthenia gravis
AChR: Acetyl-choline receptors
MuSK: Muscle-specific kinase
LEMS: Lambert-eaton myasthenic syndrome
VGCC: Voltage-gated calcium channels
ALS: Amyotrophic lateral sclerosis
FcRn: Neonatal Fc receptor
GBS: Guillain–Barre syndrome
MFS: Miller Fisher syndrome
PCB: Pharyngeal-cervical-brachial variant
SARS-CoV-2: Severe adult respiratory syndrome coronavirus 2
EMG: Electromyogram
PLEX: Plasma exchange
CT: Computer tomography
MRI: Magnetic resonance imaging
PET: Positron emission tomography
TNF: Tumor necrosis factor
SS: Sjogren’s syndrome
MS: Multiple sclerosis
NMO-SD: Neuromyelitis optica spectrum disorders
EDSS: Expanded disability status scale
FESS: Fiber endoscopic swallow studies
CSF: Cerebrospinal fluid
AQP4: Aquaporin 4
MOG: Myelin oligodendrocyte glycoprotein
SPS: Stiff-Person syndrome
PERM: Progressive encephalopathy with rigidity and myoclonus
GABAR: Gamma aminobutyric acid receptor
NMDAR: N-methyl-D-aspartate receptor
ADEM: Acute disseminated encephalomyelitis
CLIPPERS: Chronic lymphocytic inflammation with pontine perivascular enhancement responsive to steroids
DPPX: Dipeptidyl peptidase-like protein-6
GFAP: Glial fibrillary acidic protein
